# *Clostridium difficile*–associated Disease in New Jersey Hospitals, 2000–2004^1^

**DOI:** 10.3201/eid1303.060294

**Published:** 2007-03

**Authors:** Esther T. Tan, Corwin A. Robertson, Shereen Brynildsen, Eddy Bresnitz, Christina Tan, Clifford McDonald

**Affiliations:** * New Jersey Department of Health and Senior Services, Trenton, New Jersey, USA; † Centers for Disease Control and Prevention, Atlanta, Georgia, USA; 2 Current affiliation: United Nations, New York, New York, USA

**Keywords:** *Clostridium difficile*, hospital infections, surveillance, New Jersey, dispatch

## Abstract

Recent emergence of a virulent strain of *Clostridium difficile* demonstrates the importance of tracking *C. difficile* incidence locally. Our survey of New Jersey hospitals documented increases in the rates of *C. difficile* disease (by 2-fold), *C. difficile*–associated complications (by 7-fold), and *C. difficile* outbreaks (by 12-fold) during 2000–2004.

*Clostridium difficile*, a gram-positive organism, is the most common cause of nosocomial infectious diarrhea in the United States (*1*). In 2005, the Centers for Disease Control and Prevention (CDC) reported on a new, epidemic, toxin gene–variant strain of *C. difficile* on the basis of a study of isolates collected from hospitals in multiple states, including New Jersey. CDC recommended that inpatient healthcare facilities track the incidence of *C. difficile*–associated disease (CDAD), including the clinical outcomes of patients (*2*).

## The Study

To estimate the incidence of CDAD in hospitalized patients in New Jersey, we conducted a retrospective survey of acute-care hospitals. An Internet-based questionnaire was distributed to all 81 New Jersey hospitals in early 2005; hospitals that did not respond were contacted by telephone or electronic mail. We collected information on hospital characteristics, the number of CDAD cases, *C. difficile*–positive laboratory test results, *C. difficile*–associated complications, deaths due to any cause within 30 days of diagnosis with *C. difficile* infection, healthcare-associated *C. difficile* outbreaks, recurrent CDAD cases, diagnostic test methods, and surveillance activities. The proportion of community- versus healthcare-acquired cases was not assessed objectively; however, respondents provided their perceptions regarding trends. No individual patient information was obtained.

A CDAD case was defined as a patient with symptoms of diarrhea and at least 1 of the following: positive toxin assay result, diagnosis of pseudomembranous colitis on sigmoidoscopy or colonoscopy, or histopathologic diagnosis. An outbreak was defined as >3 cases of healthcare-associated CDAD in the same general area within 7 days. A complicated case was defined as a patient with CDAD in whom toxic megacolon, perforation of the colon, colectomy, or shock requiring vasopressor therapy subsequently developed within 30 days after diagnosis with CDAD. The definition of recurrent CDAD and the method of laboratory diagnoses were determined by each hospital.

Data were analyzed by using EpiInfo version 3.3.2 (CDC, Atlanta, GA, USA) and SAS version 8.02 (SAS Institute, Cary, NC, USA). The medians, means, ranges, frequencies, and totals reported are based on actual responses to the survey questions; hospitals that did not answer a question were excluded from the analysis of responses to that question. Tests for linear trend over the study period were performed by using linear regression. We also examined the association between CDAD rates and staffing levels of infection-control professionals (ICPs) by using a Poisson regression model.

Of the 81 hospitals contacted, 58 (72%), located in 20 of 21 New Jersey counties, responded to the survey. The median bed capacity of participating hospitals was 281 (range 77–683), and the median number of full-time equivalent ICPs per 250 beds was 1.2 (range 0–3).

During 2000–2004, participating hospitals reported a total of 13,394 CDAD cases. The mean annual rate of CDAD increased from 3.7/1,000 admissions in 2000 to 7.7/1,000 admissions in 2004, which represented a >2-fold increase in CDAD rates during the 5-year period (p<0.05, Table 1). Of the hospitals that responded, the percentage that did not identify CDAD cases decreased from 55% in 2000 to 25% in 2004. A significant inverse association existed between the number of ICPs per 250 beds and CDAD rates in 2004 (p = 0.05, Figure). Table 1 indicates a similar increasing trend in the rates of positive *C. difficile* test results, CDAD outbreaks, complications, annual 30-day crude mortality (deaths due to any cause), and recurrent CDAD infection. Hospitals differed widely in their case definitions for recurrent cases. Most used an arbitrary period (from first episode to CDAD relapse), varying from 6 weeks to 1 year, to define a recurrent case.

**Table 1:** *Clostridium difficile* infection rates in acute-care hospitals, New Jersey, 2000–2004*

	2000	2001	2002	2003	2004	Mean	Total	2004 rate/ 2000 rate
*C. difficile* cases	1,585	1,540	2,201	2,974	5,094	2,679	13,394	NA
*C. difficile* cases/1,000 admissions	3.7	3.2	4.1	4.9	7.7	4.7	NA	2.1
*C. difficile*–positive test results	2,355	2,759	5,193	8,592	12,445	6,269	31,344	NA
*C. difficile*–positive test results/1,000 admissions	4.2	4.4	7.8	12.4	18.4	9.4	NA	4.4
*C. difficile–*complicated cases	2	3	5	12	43	13	65	NA
*C. difficile* complication rate† (%)	0.1	0.2	0.2	0.4	0.9	0.4	NA	6.8
*C. difficile* healthcare-associated outbreaks (no. hospitals affected)	2 (2)	6 (1)	10 (7)	11 (7)	25 (12)	11	54	NA
*C. difficile* healthcare-associated outbreak rate‡	3.7	10.9	18.2	19.6	44	19.3	NA	11.9
Deaths reported within 30 days after diagnosis	0	0	23	50	87	32	160	NA
30-day *C. difficile* crude mortality rate (%)	0	0	1.2	1.9	1.8	1	NA	1.5§
Recurrent *C. difficil*e cases	0	1	7	76	171	51	255	NA
Recurrent *C. difficile* rate¶ (%)	0	0.1	0.4	2.7	3.4	1.3	NA	34#

*NA, not available. †Percentage of *C. difficile* patients in whom complications developed. ‡Outbreaks per 100 acute-care hospitals that responded. §2004 rate/2002 rate. ¶Percentage of *C. difficile* patients whose infections recurred. #2004 rate/2001 rate.

**Figure:**
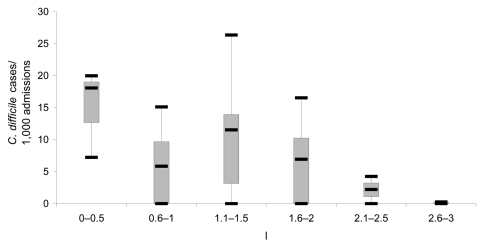
Boxplot of *Clostridium difficile* rates by number of infection-control professionals (ICPs) per 250 beds, New Jersey, 2004. Each box shows the median, quartiles, and extreme values.

Most (60%) respondents thought that the number of cases of community-acquired CDAD increased in 2004 compared with previous years. Smaller proportions of respondents perceived increases in the numbers of recurrent cases (55%), healthcare-acquired cases (40%), complicated cases (28%), and deaths (19%) during the same period.

Hospital laboratories most commonly used enzyme immunoassay (EIA) tests for toxins A and B to identify *C. difficile* (88%), followed by stool culture (16%), cytotoxin testing using tissue culture (7%), and EIA for toxin A (7%). Forty (69%) hospital laboratories reported having written institutional policies for correct specimen collection, storage, and transportation of specimens for CDAD diagnosis. Surveillance methods used by hospitals to track CDAD in their institutions are detailed in Table 2.

**Table 2:** *Clostridium difficile* surveillance activities conducted by hospitals, New Jersey, 2000–2004

Surveillance activities	No. (%) hospitals
Monitors *C. difficile*–positive laboratory results	55 (95)
Makes a distinction between community- and healthcare-acquired *C. difficile* cases	44 (76)
Uses a standard *C. difficile* case definition for surveillance	35 (60)
Monitors clinical outcome of patients with *C. difficile* infection	28 (48)
Physicians notify infection-control professional of *C. difficile* diagnoses	18 (31)

The survey design had several limitations. First, the analysis was designed to measure the overall incidence of CDAD associated with acute-care hospitalization, regardless of acquisition site, and we did not distinguish between community- and healthcare-associated infections. Second, we did not collect information on nonresponding hospitals and therefore are unable to determine if substantial differences existed between responding and nonresponding hospitals. Third, the rates of CDAD infections were calculated from data provided by the hospitals; hospitals might not have consistently followed the case definitions that were provided for reporting. We also reviewed administrative (i.e., universal billing) data as a secondary data source and found similar trends to those observed in this study. However, this process has multiple weaknesses, including ambiguities in coding and misclassification, which limit its utility for surveillance (*3*). Finally, enhanced awareness of the disease among clinicians and ICPs might have contributed to increased reporting during the 5-year period.

## Conclusions

Our results demonstrate that CDAD rates and associated complications rose rapidly among New Jersey hospitals during 2000–2004. How much of the increase reflects rising awareness and how much is a true increase in incidence is unclear. Nevertheless, the trend is dramatic and consistent with published reports in the United States, Canada, and Europe that evaluated CDAD rates during earlier periods (*4*–*7*).

Our observation that a higher ICP staffing level was associated with lower CDAD rates is consistent with previous studies demonstrating that a higher ICP-to-bed ratio is associated with reduction in rates of healthcare-acquired infections (*8*–*10*). We recommend that hospitals ensure that their infection-control programs employ sufficient personnel and other resources to implement adequate infection-control practices, with the goal of decreasing CDAD rates in their institutions.

In terms of surveillance activities, almost all participating hospitals tracked *C. difficile* laboratory results. However, a relatively low percentage of hospitals routinely monitored CDAD complications and deaths. Given recent reports of the emergence of hypertoxin-producing *C. difficile* strains that are more treatment-resistant and potentially more virulent than other strains (*2*), we recommend that hospitals implement or continue comprehensive surveillance programs to track the incidence of both healthcare-acquired and community-acquired CDAD, as well as patient outcomes. Surveillance of these entities will allow ICPs to identify quickly changes in CDAD incidence and severity that could be associated with the introduction of new, more virulent strains. In addition, rapid changes in incidence and detected outbreaks should be reported to public health officials.

Despite the survey’s limitations, the estimates provided from this substantial sample of acute-care hospitals are useful for hospitals to develop appropriate CDAD policies and can serve as comparison data for future infection prevention and control efforts in New Jersey and other states. Indeed, given the recent increase in the extent of *C. difficile* death and illness in North America and Europe, the findings in this study show that CDAD is an emerging problem, worthy of substantial investment in effective infection-control and monitoring systems.
